# Effect of body mass index and rocuronium on serum tryptase concentration during volatile general anesthesia: an observational study

**DOI:** 10.6061/clinics/2020/e1701

**Published:** 2020-08-06

**Authors:** Urszula Kosciuczuk, Pawel Knapp, Piotr Jakubow

**Affiliations:** I Department of Anesthesiology and Intensive Therapy, Medical University of Bialystok, Poland.; II Department of Gynecology and Gynecological Oncology, Medical University of Bialystok, Poland.

**Keywords:** Diagnostic, General Anesthesia, Hypersensitivity Reactions

## Abstract

**OBJECTIVE:**

Female sex, body mass index (BMI), and neuromuscular blocking agents are risk factors of perioperative hypersensitivity reactions. This study aimed to investigate the effect of rocuronium on serum tryptase concentrations during general anesthesia in overweight and obese women.

**METHODS:**

The study was conducted in two groups: Group I (n=66) underwent volatile anesthesia with rocuronium and group II (n=60) underwent volatile anesthesia without any muscle relaxant. Serum tryptase concentration (STC) measurements were performed at baseline (STC 0) and postoperatively (STC 1). ClinicalTrials.gov: NCT04035707

**RESULTS:**

The highest median value of STC 0 was seen in obese patients (3.44 μg L^-1^) and it was significantly higher than in overweight (*p*=0.01) and underweight patients (*p*=0.03). The maximum STC 0 was observed in overweight patients (20.4 μg L^-1^). In group I, STC 0 in obese patients presented the highest median value (4.49 μg L^-1^), and was significantly higher than in overweight patients (*p*=0.03), and had significantly higher STC 1 than patients with normal BMI (*p*=0.04). STC 0 and STC 1 in overweight and obese female patients did not differ significantly between groups. STC 1 did not correlate with rocuronium doses. In group I, BMI positively correlated with the duration of rocuronium infusion (rho=0.37) and STC 1 positively correlated with BMI (rho=0.32).

**CONCLUSION:**

Excess weight and obesity predispose to higher preoperative serum tryptase values. Postoperative STC is not linked to rocuronium doses. BMI is the main determinant factor of STC during combined volatile general anesthesia.

## INTRODUCTION

Perioperative hypersensitivity reactions (PHRs) are an important issue in safety and perioperative care. Epidemiological data show an increase in the incidence of hypersensitivity reactions, and they remain a life-threatening complication of general anesthesia with the mortality rate ranging from 3 to 9% (1,2). Such a trend was observed in Europe, Scandinavia, Australia, and New Zealand. The greatest risk of hypersensitivity reaction occurs during the induction phase of general anesthesia with the use of neuromuscular blocking agents (NMBa). Among many pharmacological substances used in the perioperative period, neuromuscular blocking agents are the most common cause of hypersensitivity. PHRs are estimated to occur in 1:10,000 to 1:20,000 anesthetic procedures, but the frequency increases to 1:6500 with the administration of muscle relaxants (3-5).

Many publications presented that rocuronium, a steroid non-depolarizing neuromuscular blocking agent, is a culprit trigger of PHR (6-9). The 6^th^ National Audit Project (NAP 6) identified that the overall incidence of NMBa-induced perioperative anaphylaxis was 5.3 per 100,000 exposures, and anaphylaxis rate connected with rocuronium administration was 5.8 per 100,000 administrations (6-10).

PHRs to NMBa do not have a uniform pathogenesis. The sensitization phenomenon in the immunological mechanism is associated with cellular and humoral response after the first contact with the allergen, and leads to the synthesis of specific immunoglobulin E (IgE), which remains linked to high affinity for FcγRI receptors. Another potential mechanism of PHR is the non-immunological mast cell activation with anaphylatoxins C3a and C5a via specific receptors. Recent publications have described that NMBa can activate mastocytes through the MRGPRX 2 receptor (MAS-related G protein-coupled receptor member X2) (11-15).

Tryptase is the main mediator secreted by activated mast cells in hypersensitivity reactions. Serum tryptase concentration (STC) in the normal state ranges between 1 and 11.4 µg L^-1^. The measurement of serum tryptase is interdisciplinary, and is recommended as a basic element in the diagnosis of perioperative hypersensitivity events (16-21).

In many epidemiological publications, the anthropometric risk factors of PHR were described. Excess weight and obesity were presented as a common state in PHRs, and other risk factors like sex and age were also described. The ratio of the PHRs in female to male patients is 3:1. In the adult population, PHR is more frequent in women of about 40 years and in men over 50 years of age (22-25).

The current study is a clinical observational investigation that evaluates the effect of rocuronium administration during volatile general anesthesia in the female population on hypersensitivity reaction induction. We used serum tryptase measurements to test the following hypotheses: rocuronium is a risk factor of general anesthesia-induced hypersensitivity reactions, and body mass index (BMI) determines the perioperative serum tryptase concentration.

## METHODS

This observational study was approved by the Bioethical Committee (R-I-002/286/2009), supported by the grant of the Medical University of Bialystok (143-14548/14), and registered in ClinicalTrials.gov (NCT04035707). Written informed consent was obtained from all patients.

We divided 126 female patients over the age of 18 years classified in ASA 1-2, without allergy and PHRs into two groups according to the surgical qualification.

- **group I:** 66 patients qualifying for the gynecologic operation procedures underwent volatile general anesthesia with the muscle relaxant (rocuronium)
- **group II:** 60 patients qualifying for thyroidectomy underwent volatile general anesthesia without muscle relaxant

The exclusion criteria were as follows: a medical history of allergy, steroid therapy, mastocytosis, and hypersensitivity reactions.

In both groups, we performed volatile induction and maintenance of anesthesia with sevoflurane (Sevorane, Abbvie). During the induction of anesthesia, we assessed patients in group I after an adequate level of anesthetic sleep, we administered a neuromuscular blocking agent, rocuronium (Esmeron, Organon), at a dose of 0.6 mg/kg and we started monitoring for muscle relaxation with the use of train-of-four (TOF) stimulation. Tracheal intubation was performed when TOF 0 was assessed. When muscle relaxation returned to TOF 4, continuous infusion of rocuronium at a speed required to achieve TOF 0 was administered, and stopped at the surgical closing of the peritoneal cavity. After the respiratory movement appeared and neuromuscular function returned to the level of TOF 25%, 0.5 mg of atropine (Atropinum sulfuricum, Polfa) and 1.5 mg of neostigmine (Polstygmina, Pliva) were administered intravenously. Extubation was performed in hemodynamic stable patients with spontaneous respiratory function and TOF ratio of 0.8-0.9.

In group II, because of the surgical neuroidentification and stimulation of the laryngeal recurrence nerves during thyroid surgery, no NMBa was used. After an adequate level of anesthetic sleep, tracheal intubation was performed.

In both groups, analgesia was ensured through the administration of intravenous fractional doses of fentanyl at 2 µg kg^-1^ (Fentanyl, Polfa) in the induction and maintenance phase of anesthesia. After the end of the surgical procedure, the administration of sevoflurane was stopped and access to fresh gases was increased.

In all patients, anthropometric data, the duration of anesthesia, duration of surgery, volume of the applied fluid therapy, and total dose of opioids were noted. In the study group, the following values associated with rocuronium administration were noted: the intubation dose, infusion dose, total dose, and duration of infusion. In all patients, blood samples before and after anesthesia were taken for the determination of serum tryptase concentration. Immunofluoroenzymatic tests (UniCap Tryptase, Pharmacia Diagnostics) were used.

### Statistical analysis

Statistical analysis was performed with the use of STATISTICA12.0. The data were assessed for normality using the Shapiro-Wilk test. Since the data were not normally distributed, the values were quoted as median, minimum and maximum, and interquartile ranges (IQR). Sample size (n) is indicated in the figure legends. To compare variables, the following tests were used where appropriate: Wilcoxon test, and Mann-Whitney *U*-test, Kruskal-Wallis test. The Spearman rank-correlation test was used for the assessment of correlations. Correlations were shown by means of the Spearman coefficient. A *p* value of 0.05 or less indicated a significant difference.

## RESULTS

The characteristic data of the study groups are presented in Table 1. Both groups did not differ significantly in the anthropometric and clinical parameters. In the total study group, overweight and obese female patients constituted 57% and 14%, respectively while in groups I and II, overweight and obese female patients accounted for 66% and 76% of the patients respectively.

**Figure 1:**
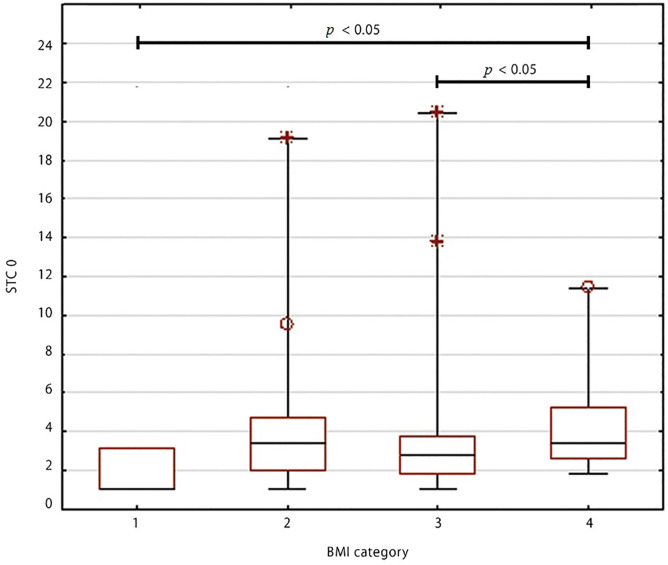
Baseline serum tryptase concentration per BMI category. Median, minimum and maximum, interquartile ranges, outliers, and extreme values are presented.

The highest median values of baseline STC (STC 0) were noticed in obese patients [3.44 μg L^-1^, IQR: 2.65-5.28) μg L^-1^] and it was significantly higher than those in overweight [median 2.75 (IQR: 1.8-3.7), μg L^-1^] (*p*=0.01) and underweight female patients [median 1.0 (IQR: 1.0-3.13), μg L^-1^], (*p*=0.03). The maximum values of STC 0 were observed in overweight patients (20.4 μg L^-1^). The STC 0 per BMI category are presented in Figure 1. 

In group I, the highest median value of STC 0 [median 4.49 (IQR: 2.45-6.54), μg L^-1^] was noted in obese patients, and it was significantly higher than that in overweight patients [median 2.66 (IQR: 1.83-4.10), μg L^-1^] (*p*=0.03). Moreover, obese patients had significantly higher postoperative STCs [median 3.56 (IQR: 2.0-6.56), μg L^-1^] than patients with normal BMI [median 1.76 (IQR: 1.0-3.84), μg L^-1^] (*p*=0.04). In these BMI categories, serum tryptase levels were higher before anesthesia and were lower after anesthesia. Postoperative serum tryptase values presented a specific trend - the lowest values were observed in patients with normal BMI, and the highest in obese patients. An analysis showed a decreased level of this enzyme, which reached 35% in the normal BMI category and 21% in obese patients, and the lowest change of its value was noticed in overweight patients - only 1%.

**Figure 2:**
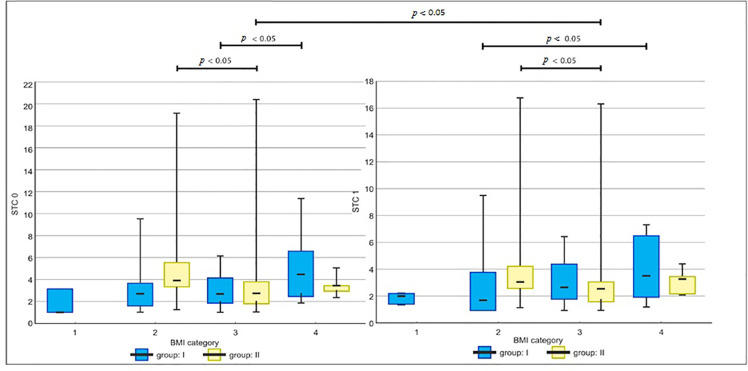
Baseline and postoperative serum tryptase concentration in the study groups. Median, minimum and maximum, and interquartile ranges are presented.

In the group of patients undergoing general anesthesia without the use of rocuronium, STC 1 decreased in a similar manner, with the highest value in the normal BMI category (20%), and the lowest reduction observed in overweight and obese patients, 5% and 2%, respectively. Patients with normal BMI ranges had significantly higher STC 0 [median 3.86 (IQR: 3.29-5.54), μg L^-1^], and STC 1 [median 3.10 (IQR: 2.66-4.28), μg L^-1^] values than overweight patients - STC 0 [median 2.75 (IQR: 1.81-3.75), μg L^-1^] (*p*=0.007) and STC 1 values [median 2.63 (IQR: 1.65-3.13) μg L^-1^] (*p*=0.03). The baseline and postoperative STC in overweight and obese females did not differ significantly between groups, but STC 1 in obese patients reached the highest median values in the study groups, in group I [median 3.56 (IQR 2.0-6.56), μg L^-1^] and in group II [median 3.38 (IQR: 2.2-4.42), μg L^-1^]. We found a non-significant reduction in postoperative STC in obese patients in both groups, and a significant decrease in overweight females in group II, STC 0 [median 2.75 (IQR 1.81-3.75), μg L^-1^] and STC 1 [median 2.63 (IQR 1.65-3.13), μg L^-1^] (*p*=0.0002). The baseline and postoperative STC in the study groups are presented in Figure 2.

STC 1 did not present any correlation with the rocuronium administration parameters: the intubating dose, the infusing dose, the total dose, and perioperative fluid therapy. In group I, the BMI positively correlated with the duration of rocuronium infusion. In addition, postoperative serum tryptase concentration in group I positively correlated with BMI value.

## DISCUSSION

The topic of PHRs is very current and the knowledge about this issue has grown significantly over the past decades. The initial reports presented that PHR associated with the use of neuromuscular blocking agents occurred in approximately 50-70% of cases (6-8,12,17). Other pharmacological substances (opioid, intravenous anesthetics, local anesthetics) were less important and accounted for approximately 4%. Long-term observations showed a reduction in the occurrence of hypersensitivity reactions related to latex (26-29). Dominance of muscle relaxants in the induction of PHRs is a characteristic and permanent phenomenon in European and Scandinavian countries (1,3,4,10). Data were from the American population indicated that the main factors causing hypersensitivity were antibiotics, especially penicillin and cephalosporin, which constituted up to 50% of cases, while neuromuscular blocking agents constituted only 10% of etiological factors (30).

The specificity of general anesthesia limits the possibilities of hypersensitivity reactions diagnosis. The authors of the NAP 6 presented that the most frequently observed symptoms were hypotension (46%), bronchospasm (18%), tachycardia (9%), bradycardia (3%), oxygen desaturation (4.8%), and reduced or absent capnography trace. It was described that 58% of severe PHRs occurred in the operating theater area, and 3% of them happened before the induction of anesthesia and 81% in the period after induction but before surgery, 13% during the surgical procedure and 3% after surgery (10). Berroa et al. describing hypersensitivity reactions associated with general anesthesia showed that the use of NMBa in the induction phase increased the frequency of their occurrence from 1: 1600 to 1: 6600 procedures (31). The severity of clinical symptoms of PHR can be assessed by many classifications, but the first classification was the Ring and Messmer Scale. Modern classifications combine the clinical signs and results of biochemical measurements of serum tryptase concentration (5).

Numerous publications presented that rocuronium is the most common factor of hypersensitivity reactions; in 52% of cases they were related to steroid NMBa, including 55% of cases linked to rocuronium. The incidence of hypersensitivity reactions during general anesthesia resulting from the use of suxamethonium and benzylisoquinoline derivatives was equal (6-8,32). The incidence of anaphylaxis associated with non-depolarizing muscle relaxants was significantly higher with rocuronium, and was 8 cases/100,000 applications/10 years, for vecuronium 2/100,000/10 years, and for atracurium 4/100,000/10 years (33,34).

The main diagnostic methods of hypersensitivity reactions during general anesthesia are the determinants of serum tryptase concentration. Recently, the maximum ranges of 11.4 µg L^-1^ have been abandoned and the >2+1.2×baseline tryptase algorithm is suggested. Baseline blood samples should be taken a minimum of 24 hours after the event appearance and can be performed postmortem, because of high chemical stability (1-4).

The diagnosis of PHR is difficult, and it has been reported that in 30% of PHR cases, the etiologic factors ware not confirmed (35). In 82% of patients who experienced PHR associated with a specific muscle relaxant, cross-reactions in the same chemical group were found. Among patients tested for rocuronium-induced hypersensitivity reactions, 44% of cases showed a positive skin test with suxamethonium, 40% of cases with vecuronium, and 20% of cases with pancuronium and atracurium (33).

Some studies have presented that cross-reactions and sensitization with NMBa were connected with environmental factors (9,10,12,16). Both pholcodine, an antitussive agent, and chemicals and cosmetics containing quaternary ammonium group were able to induce IgE production and sensitization state. In the light of this information, the first exposure to NMBa in patients without a medical history of allergy may provoke perioperative hypersensitivity reaction (3,4).

There are not many publications on changes in serum tryptase concentration during general anesthesia. It was described that preoperative serum tryptase concentration in the orthopedic population was 5.01 μg L^-1^ and was significantly reduced after general anesthesia and surgery. The authors emphasized the influence of fluid therapy on serum tryptase concentration. Numerous researchers have shown that there is a dilution effect of crystalloid fluid therapy in the determination of hemoglobin and hematocrit, but no studies have been conducted describing the effect of intraoperative fluid therapy on the determination of serum tryptase values (36). In order to limit the effect of dilution in blood sampling we used a separate peripheral intravenous route.

Many authors pointed a correlation between STC and anthropometric parameters, as well as co-morbidities. In individuals above 60 years, the mean serum tryptase concentration was 13 μg L^-1^ and it was not associated with the appearance of hypersensitivity symptoms (3,5,8,10,37). Another study indicated that the median STC in a group of patients aged between 18 and 30 years was the lowest at 4 μg L^-1^, while a significant increase in the median value to 6.6 μg L^-1^ was noted in the age groups above 50 years (8). Individual variability in enzyme concentration was found in healthy patients at various time intervals, with an average of 0.26 μg L^-1^ (21). STC was significantly higher in overweight and obese patients, simultaneously emphasizing the dependence of elevated STC on occurrence of metabolic syndrome (38). The NAP 6 described that 21% cases of deaths of complicated severe PHR were in obese and morbidly obese patients (10).

Particular perioperative attention should be given to patients with mastocytosis, because mast cell dysfunction leads to hypersensitivity to drugs and agents used during general anesthesia. In this group of patients, propofol, etomidate, ketamine, sevoflurane, desflurane, midazolam, fentanyl and its derivatives, paracetamol, amide local anesthetics and non-depolarizing steroids, and benzylisoquinoline neuromuscular blocking agents are considered safe substances (39,40). In the selection of a group of patients, we excluded patients with mastocytosis.

The main source of information on epidemiological factors and the course of PHRs are retrospective analyses. We decided to evaluate the effect of rocuronium on the STC in the female population with further analysis into overweight and obese patients. We included three risk factors in the study: female sex, chemical substance-rocuronium, and BMI. Despite the limited study group, the obtained results served as important and new information that BMI, and not rocuronium, is the main determinant factor of serum tryptase concentration during combined volatile general anesthesia. In order to describe the exact effect of relaxants on the serum tryptase concentration and assess the risk of hypersensitivity reactions induction, this scheme of study should be performed with other substances.

## CONCLUSIONS

1. Excess weight and obesity predispose to higher preoperative serum tryptase values.
2. The use of rocuronium during combined volatile general anesthesia in overweight and obese female patients did not result in specific changes in STC compared to volatile induction and maintenance of anesthesia without neuromuscular blocking agents.
3. Postoperative STC is not directly connected with rocuronium doses, but is connected with BMI values during general anesthesia with effects on the postoperative STC.

## AUTHOR CONTRIBUTIONS

Kosciuczuk U conceived and designed the study, was also responsible for the data acquisition, analysis and interpretation, and manuscript drafting. Knapp P designed the study and was responsible for the manuscript critical revision. Jakubow P was responsible for the manuscript revision and translational corrections.

## Figures and Tables

**Table 1:** Patients’ characteristics and anthropometric data.

	Group I	Group II
Age (years)	47.5 (30-69)	49.3 (23-76)
Weight (kg)	75 (46-120)	82 (50-125)
Body mass index (BMI) (kg m^-2^)	26.4 (18.2-37.3)	26.2 (18.5-37.4)
Body surface area (BSA) (m^2^)	1.87 (1.42-2.37)	1.76 (1.46-2.38)
BMI categories 1/2/3/4 (n)	3/19/32/12	0/14/40/6
Duration of surgery (min)	95 (45-190)	86 (53-160)
Duration of general anesthesia (min)	110 (60-205)	123 (70-180)
Perioperative fluid therapy (ml)	1200 (750-1850)	1250 (800-2300)
Total IgE (kU L^-1^)	61.3 (2.72-112.0)	58.5 (2.14 -99.0)

Median, minimum and maximum ranges are presented.

BMI categories:

1- underweight BMI<18.49; 2- normal BMI 18.5-24.99;

3- overweight BMI 25-29.99; 4- obesity BMI>30.
